# Factors associated with cognitive function outcomes among older adults in Kuwait: A cross-sectional study

**DOI:** 10.1186/s12877-025-05882-0

**Published:** 2025-04-14

**Authors:** Thurayya Albuloshi, Ahmed M. Kamel, Ahmad R. Alsaber, Balqees Alawadhi, Jiazhu Pan, Wafaa Mostafa Abd-El-Gawad, Manal Bouhaimed, Jeremy P.E. Spencer

**Affiliations:** 1 Palliative Care Center, Kuwait, Ministry of Health, Al Sabah Medical Area, P.O. Box 5, Kuwait City, 13001 Kuwait; 2 Department of Clinical Pharmacy, Faculty of Pharmacy, Cairo University, Kasr El-Aini, Cairo, 11562 Egypt; 3 Department of Management, College of Business and Economics, American University of Kuwait, 15 Salem Al Mubarak St, Salmiya, Kuwait; 4 Faculty of Health Sciences, The Public Authority for Applied Education Training, Shuwaikh Industrial, Kuwait; 5 Department of Mathematics and Statistics, Faculty of Science, University of Strathclyde, 26 Richmond, Glasgow, G1 1XH UK; 6 Department of Geriatrics and Gerontology, Faculty of Medicine, Ain Shams University, Al-Abbasseya, Cairo, Egypt; 7 Department of Community Medicine and Behavioral Sciences, Faculty of Medicine, Kuwait University, P.O. Box 24923, Safat, 13110 Kuwait; 8 Hugh Sinclair, Unit of Human Nutrition, Department of Food and Nutritional Sciences, School of Chemistry, Food and Pharmacy, University of Reading, Reading, RG6 6AP UK

**Keywords:** Older adults, Cognitive impairment, Type 2 diabetes, Hypertension, Aging, Kuwait

## Abstract

**Background:**

The number of people living with dementia and/or cognitive impairment worldwide is rising with a negative effect on quality of life for many older adults. This study aims to examine the factors associated with cognitive function among older adults in Kuwait.

**Methods:**

This cross-sectional study recruited 253 older adults ≥ 60 years from a Geriatric outpatient unit in Kuwait. Cognitive function (dependent variable) was assessed using the Arabic version of the Mini-Mental State Examination (MMSE) with scores < 24 indicative of cognitive impairment. Biochemical, nutritional, clinical, lifestyle, anthropometric, and sociodemographic independent variables were included.

**Results:**

A normal MMSE score was reported for 51.0% (*n* = 129) of the sample, with 34.7% and 14.2% of participants having mild and moderate/severe cognitive impairment, respectively. Multivariate ordinal logistic regression analysis indicated that Type 2 diabetes was associated with more than double the odds of cognitive impairment (OR = 2.15, 95% CI: 1.19–3.94; *P* = 0.01). Each additional level of education was associated with a lower likelihood of cognitive impairment (OR = 0.34, 95% CI: 0.26–0.43; *P* < 0.001).

**Conclusion:**

This study identifies key risk factors associated with cognitive impairment in older Kuwaiti adults. These findings underscore the need for targeted interventions to mitigate cognitive decline in aging populations and provide context-specific data to support policy decisions.

**Supplementary Information:**

The online version contains supplementary material available at 10.1186/s12877-025-05882-0.

## Introduction

The proportion of individuals aged 60 years and above in the global population is currently at 12%, with projections indicating a rise to 22% by 2050.Research also suggests a 56% increase in the global population of those aged 60 years and older from 2015 to 2030, and those over 65 years are expected to constitute over half of the world’s population by 2030. These trends are also apparent in Kuwait. As of 2023, individuals aged 60 and over constitute approximately 7.6% of Kuwait’s total population, which is projected to rise to 39.6% by 2050 [1]. The aging of populations is a key factor contributing to shifts in morbidity and mortality patterns, leading to a higher prevalence of non-communicable diseases, including mental and neurological disorders [2].Mental and neurological conditions impact over 20% of the elderly population globally, with dementia being one of the most prevalent disorders affecting around 5% of this demographic [3].Apart from the direct health implications, mental health disorders also have significant economic consequences [4, 5].

Currently, there are 50 million individuals globally living with cognitive impairment, a number projected to surpass 150 million by 2050 in the absence of effective treatments [6]. The burden of cognitive impairment in older individuals is anticipated to rise in Kuwait as the population of those over 60 years old grows. Identifying the risk factors for cognitive decline is crucial for implementing targeted interventions to prolong independence and delay the onset of dementia [7].

While ageing is a significant factor in cognitive function deterioration [8], there are modifiable factors linked to cognitive impairment [9]. Research is increasingly uncovering evidence on dementia risk factors, with the most robust associations found for aging, low educational attainment in early life, diabetes, midlife hypertension, genetic factors like the APOE gene, cardiovascular disease, dyslipidemia, psychiatric conditions such as depression, and obesity [10–13].

In addition, certain lifestyle factors could enhance or introduce the risk of cognitive decline (for example, alcohol consumption and smoking) [14, 15]. In contrast, physical activity, cognitive stimulation, a high level of education, and healthy dietary patterns are identified as protective factors against cognitive impairment [9, 16]. There is also some evidence to support a connection between cognitive decline and cortisol [17–19] and alkaline phosphatase [20]. In the Kuwaiti population, reported risk factors for cognitive impairment include obesity, which adult Kuwaitis rank among the highest in the world, with 63% of the Kuwaiti population aged 60–69 years classified as obese [21]). Nevertheless, in Kuwait and across the other Gulf countries, studies examining the factors associated with cognitive decline in older adults are lacking. Therefore, this study examines the factors associated with cognitive function outcomes among older adults (≥ 60 years) in Kuwait, including the association between comorbidities, laboratory test results, anthropometric measurements, nutritional status, lifestyle factors, and cognitive function among older adults aged 60 years and above in Kuwait. A multifactorial assessment of cognitive function is essential for public health planning in Kuwait. With the ageing population, gaining a better understanding of the modifiable and non-modifiable risk factors of cognitive decline is crucial to inform early intervention and preventive strategies by the Ministry of Health and other stakeholders. By identifying population-specific risk and protective factors for cognitive impairment, this study provides essential context-specific evidence that policymakers can use to garner support for targeted interventions in Kuwait.

## Methods

Broadly, this study involves the administration of a number of questionnaires to patients to capture data on cognitive performance and risk factors, along with laboratory measures of biochemical variables.

### Population and study design

This was a nationally-representative study employed a convenience sampling approach and collected between November 2020 and June 2021. Participants were selected from seven primary healthcare centres with geriatric clinics. In brief, eligible participants were Kuwaiti individuals aged 60 years or older, of any gender, without disabilities, willing to participate, providing complete data, and capable of undergoing assessments without hearing, vision, or speech impairments. In regard to living arrangements, most of the older adults who participated in this study live independently in their own homes, often with their children. Only a few participants rent their homes, and none of them reside in an elder house.Potential sources of selection bias were addressed by implementing random sampling procedures and stratification based on key demographic variables. Of the 332 individuals approached, 79 declined to participate, 253 agreed to participate, and 253 completed the study. The individuals who opted not to participate or who were lost to attrition did not differ in their demographic profile compared to those who participated.

The entire questionnaire underwent validation and pilot testing with a group of 10 volunteers who were not part of the main research project.

### Sociodemographic and lifestyle variables

Socioeconomic variables collected by interviews include age, gender, marital status (i.e., married, divorced, single widowed), monthly income (i.e., 500–1000 K.D., 1001–1500 K.D., 1501–2000 K.D., > 2000 K.D.), and educational level (i.e., No formal education, Completed primary/intermediate school, Completed secondary school, Completed Diploma, University degree or higher). Self-reported lifestyle factors collected included smoking status (yes/no), alcohol consumption (yes/no), sleep duration (hours per day) (≥ 9 h, 7–8 h, and ≤ 6 h), Physical activity (yes/no), minutes of walking per day, and sun exposure per day (< 5 min, 5–30 min). Physical activity was evaluated using the International Physical Activity Questionnaire for the Elderly (IPAQ) [22], known for its established validity and reliability in Arabic [23].

### Assessment of cognitive function

 The MMSE is a copyrighted instrument and may not be used or reproduced in whole or in part, in any form or language, or by any means without written permission of PAR (www.parinc.com). The Mini-Mental State Examination (MMSE) is a widely recognized 30-item questionnaire employed for evaluating cognitive impairment in both clinical and research settings [24]. This assessment tool comprises 11 items designed to assess five key cognitive domains: orientation, registration, attention and calculation, recall, and language. It takes just 7–10 min to complete, making it practical for repeated and routine use. Globally, the MMSE is one of the most frequently used instruments for screening cognitive function. Localized versions of the MMSE have been developed to account for the variation in scores based on cultural and sociodemographic variables. In the absence of a local validation of the MMSE for Kuwait, the validated Arabic version of the MMSE was used in this study. In the original MMSE scoring system, a score below 24 indicates cognitive impairment, with a maximum possible score of 30 representing no cognitive impairment. However, to improve accuracy, recent research has adjusted these cutoff points based on the educational level of the individual. According to these updated guidelines, a score of 24–30 is categorized as normal; a score of 19–23 suggests mild cognitive impairment; a score of 10–18 indicates moderate cognitive impairment; and a score of 0–9 is classified as severe cognitive impairment [25, 26]. A score of 19 and below, when adjusted for education, demonstrates a sensitivity of 60.9% and specificity of 59.5% [27]. An unauthorized version of the Arabic MMSE was used by the study team without permission, however this has now been rectified with Psychological Assessment Resources (PAR).

### Nutrition variables

Calcium intake, vitamin D intake, and vitamin D supplementation details were collected through structured interviews using a previously validated version of a semi-quantitative Food Frequency Questionnaire (SFFQ). To enhance the rigor and accuracy of the reported outcomes, trained interviewers followed a standardized protocol, and responses were cross-verified for consistency [28–30]. NutriBase Pro 19 software was utilized to analyze the nutrient content of different foods, with reference to the ‘Food composition database: Kuwaiti composite dishes’ for traditional Kuwaiti meals [31].

### Anthropometric variables

Anthropometric measurements included height, weight, body mass index (BMI), categorized based on WHO guidelines into normal weight (18.5–24.9 kg/m^2^), overweight (25.0–29.9 kg/m^2^), and obese (≥ 30 kg/m^2^) [32],waist circumference and risk (low, high), and Waist-hip ratio (WHR) and risk (normal, at risk). WHR was calculated following WHO recommendations, with elevated WHR risk defined as ≥ 0.85 for women and ≥ 0.90 for men [32].High risk for waist circumference was considered as ≥ 80 cm for women and ≥ 94 cm for men [33, 34]. Height, weight, and waist-hip measurements were obtained using standardized measurement protocols. Calibrated equipment, including a stadiometer for height, a scale for weight, and a measuring tape for waist and hip circumference, was used. Consistent procedures were followed for each measurement to ensure accuracy and minimize variability.

### Clinical variables

Clinical variables obtained from participants’ medical charts included previous diagnoses of comorbidities (hypertension, type 2 diabetes, cardiovascular disease, dyslipidemia, thyroid disorder, and rheumatoid arthritis). Referring medical professionals confirmed the participants’ medical history. Blood pressure (systolic and diastolic) was measured using a mercury sphygmomanometer on the left upper arm [28],following the International Society of Hypertension Global Hypertension Practice Guidelines [35]. Elevated blood pressure was defined as [elevated blood pressure was defined as a systolic blood pressure (SBP) of ≥ 130 mmHg or a diastolic blood pressure (DBP) of ≥ 80 mmHg ] (International Society of Hypertension, 2020) [35].

Depressive symptoms were assessed by a geriatrician using the 15-item Geriatric Depression Scale (GDS-15), a tool developed by Yesavage et al. [36] to evaluate depression symptoms in older populations. The GDS-15 has previously been validated for clinical use and translated into Arabic [37, 38]. We applied the Arabic version of the GDS-15 for this study. It consists of 15 questions, with affirmative responses to 10 items indicating depression, while negative responses to questions 1, 5, 7, 11, and 13 suggest depressive symptoms. Scores ranging from 0 to 4 are considered normal, 5–8 indicate mild depression, 9–11 signify moderate depression, and 12–15 indicate severe depression [36]. A score of 8 or higher is the most reliable threshold for identifying clinically significant depression, with a high level of reliability [39]. We administered the GDS-15 as an interview questionnaire with researchers verbally presenting each question to participants and recording their responses [Interviews were conducted by T.A. following a standardized protocol to ensure consistency and accuracy in data collection].

Pigmentary phototype was assessed using Fitzpatrick Classification of Skin Phototype was applied by one assessor (for consistency) to measure the color of the participants’ skin as (I) very fair, (II) fair, (III) fair to medium, (IV) medium, (V) olive or dark, and (VI) very dark with deep pigmentation] the Fitzpatrick scale [28].

### Biochemical variables

Several biochemical tests were conducted, including for fasting blood glucose, insulin, cortisol, alkaline phosphatase, serum phosphorus, calcium, serum vitamin D (25-OH-D), and parathyroid hormone to capture. were measured to capture key biomarkers that may influence metabolic, endocrine, and bone health, all of which have been implicated in cognitive function and aging in older adults. A trained nurse extracted 10 mL of blood from each study participant using a gel-based tube (SST II Advance, BD Vacutainer) for collection. The measurement of serum cortisol was carried out using electrochemiluminescence on an e411/ELECSYS instrument, with blood samples taken early in the morning between 7:00 and 8:00. Fasting blood glucose levels were analyzed with the Architect c4000 clinical chemistry system. Serum calcium concentrations were determined utilizing the Arsenazo III dye method (Architect plus c 4000, Abbott, Abbott Park, IL, USA). Insulin levels were assessed by here the chemiluminescent immunoassay (CLIA) method using the IMMULITE 2000XPi system (Siemens Healthineers, Erlangen, Germany). The analysis of serum phosphorus concentrations and alkaline phosphatase activity was performed using the phosphomolybdate UV method on the same system. During handling and storage to prevent degradation from light, serum samples for vitamin D analysis were kept in the dark, with plasma stored at -80 °C until it was analyzed. Plasma 25-OH-D concentrations were determined using liquid chromatography tandem mass spectrometry (LC/MS/MS) in a laboratory accredited by the College of American Pathologists.

### Ethical consideration

The research protocol was reviewed and approved by the Kuwait Ministry of Health Standing Committee for the Co-ordination of Medical Research (approval protocol no.: 2019/1016) and the University of Reading Ethics Committee for Clinical Research (approval protocol no.: URCE 19/47), in compliance with the guidelines of the Declaration of Helsinki. Each participant received detailed information about the study [28], including its objectives and procedures. Risks and benefits were clearly outlined in the consent forms signed by willing participants.

### Statistical analysis

The sample size was calculated using G*Power software (3.2.1 version) for a multivariate original logistic regression model assessing the association between cognitive impairment and key predictors. Effect sizes were derived from previous literature on cognitive impairment risk factors using the weakest predictor. The sample size was determined using an assumed effect size based on prior studies, with a power of 80%, an alpha level of 0.05, and a confidence interval of 95%. The minimum population sample size for this study was determined to be ensure the completeness of the data and minimize missing information, additional calculations were performed beyond the minimum required for analysis. This was done to handle any missing data effectively and improve the reliability of our results.

Categorical data were presented through frequency counts and percentages [40].For variables following a normal distribution, the mean and standard deviation (SD) were used for summary, while the median and interquartile range (IQR) were employed for variables with non-parametric distribution. To assess relationships between categorical variables, the chi-square independence test was utilized [41].Comparisons of mean values across groups for variables with normal distribution were carried out using the two-sample t-test and one-way Analysis of Variance (ANOVA). The Mann-Whitney U test and the Kruskal-Wallis test were applied to analyze variables with a non-normal distribution.

We estimated our required sample size using two approaches. Based on an approximate 30% prevalence of mild cognitive impairment among adults aged ≥ 60 years reported in global epidemiologic studies, a single-population proportion formula indicated that we needed at least 224 participants to achieve a 95% confidence interval with ± 6% precision. Allowing for 10% non-response or missing data, the target was approximately 246 participants.

The univariate testing was also stratified by gender because men and women may differ in both the prevalence and the underlying risk factors of cognitive impairment. Biological differences and sociocultural factors could modify the relationship between various predictors and cognitive outcomes. Binary logistic regression model was fitted to assess the independent predictors of cognitive impairment. The initial model included gender, age, body mass index category, education, physical activity, depressive symptoms, sun exposure category, alkaline phosphatase level, cortisol category, dietary calcium intake, diabetes status, dyslipidemia, and hypertension. Backward stepwise elimination was then conducted using the Akaike Information Criterion (AIC) as the selection metric, iteratively removing variables that did not improve model fit (*p* > 0.05). This process continued until the final parsimonious model was reached (lowest AIC, *p* < 0.05). The final model include age, education, and diabetes, each retaining a statistically significant association with the binary MMSE outcome (*p* < 0.05). The % of missing data did not exceed 2% for any of the included variables and were imputed prior to analysis. Statistical evaluation was performed with R software [42],version 4.3. Hypothesis testing was performed at a 5% level of significance.

## Results

Several sociodemographic, lifestyle, and clinical variables were significantly associated with abnormal MMSE scores (Table 1). Older age was higher in individuals with abnormal MMSE scores compared to those with normal scores (72.8 [68.7;76.1] vs. 69.1 [66.9;72.4], *p* < 0.001). Gender distribution also showed a significant difference, with a higher proportion of males in the normal MMSE group (78.3%) compared to the abnormal group (21.7%), while females were more evenly distributed (60.9% vs. 39.1%, *p* = 0.005). Education level was significantly related to cognitive impairment, with the highest percentage of abnormal MMSE scores found in those with no formal education (77.4%) and the lowest in those with a diploma (6.38%, *p* < 0.001).

**Table 1:** Sociodemographic characteristics stratified by cognitive impairment

	Overall	Normal cognitoin	Cognitive impairment	*P*
	*N* = 253	*N* = 174	*N* = 79	
**SOCIODEMOGRAPHIC VARIABLES**				
**Age in years**	69.7 [67.1;74.1]	69.1 [66.9;72.4]	72.8 [68.7;76.1]	**< 0.001**
**Gender**,** n(%)**:				**0.005**
Female	138 (54.5%)	84 (60.9%)	54 (39.1%)	
Male	115 (45.5%)	90 (78.3%)	25 (21.7%)	
**Marital status**,** n(%)**:				**< 0.001**
Married	188 (74.3%)	143 (76.1%)	45 (23.9%)	
Divorced	8 (3.16%)	6 (75.0%)	2 (25.0%)	
Single	4 (1.58%)	3 (75.0%)	1 (25.0%)	
Widowed	53 (20.9%)	22 (41.5%)	31 (58.5%)	
**Education level**,** n(%)**:				**< 0.001**
No formal education	53 (20.9%)	12 (22.6%)	41 (77.4%)	
Completed primary/intermediate school	38 (15.0%)	29 (76.3%)	9 (23.7%)	
Completed secondary school	58 (22.9%)	43 (74.1%)	15 (25.9%)	
Completed Diploma	47 (18.6%)	44 (93.6%)	3 (6.38%)	
University degree or higher	57 (22.5%)	46 (80.7%)	11 (19.3%)	
**Income per month**,** n (%)**:				**0.001**
500–1000 K.D.	118 (46.6%)	68 (57.6%)	50 (42.4%)	
1001–1500 K.D.	59 (23.3%)	41 (69.5%)	18 (30.5%)	
1501–2000 K.D.	43 (17.0%)	36 (83.7%)	7 (16.3%)	
>2000 K.D.	33 (13.0%)	29 (87.9%)	4 (12.1%)	
**LIFESTYLE & NUTRITION VARIABLES**				
**Smoking cigarettes (yes/no)**,** n(%)**:				0.461
No	234 (92.5%)	159 (67.9%)	75 (32.1%)	
Yes	19 (7.51%)	15 (78.9%)	4 (21.1%)	
**Drinking alcohol (yes/no)**,** n(%)**	4 (1.58%)	4 (100%)	0 (0.00%)	0.313
**Dietary intake of vitamin D (IU)**	190 [113;286]	192 [113;298]	184 [109;255]	0.415
**Vitamin D suppl. (yes/no)**,** n(%)**:				1.000
No	148 (58.5%)	102 (68.9%)	46 (31.1%)	
Yes	105 (41.5%)	72 (68.6%)	33 (31.4%)	
**Dietary intake of calcium (mg)**	190 [113;286]	192 [113;298]	184 [109;255]	0.415
**Calcium suppl. (yes/no)**,** n(%)**:				0.440
No	246 (97.2%)	168 (68.3%)	78 (31.7%)	
Yes	7 (2.77%)	6 (85.7%)	1 (14.3%)	
**Walk days per week**	4.00 [0.00;7.00]	4.00 [0.00;7.00]	0.00 [0.00;6.00]	0.097
**Physical activity**,** n(%)**:				**0.048**
No	137 (54.2%)	80 (58.4%)	43 (31.4%)	
Yes	116 (45.8%)	49 (42.2%)	45 (38.8%)	
**Sun exposure category**,** n(%)**:				0.254
<5 min	231 (91.3%)	156 (67.5%)	75 (32.5%)	
5–30 min	22 (8.70%)	18 (81.8%)	4 (18.2%)	
**Pigmentary phototype**,** n(%)**:				0.356
II	3 (1.19%)	1 (33.3%)	2 (66.7%)	
III	62 (24.5%)	44 (71.0%)	18 (29.0%)	
IV	161 (63.6%)	108 (67.1%)	53 (32.9%)	
V	27 (10.7%)	21 (77.8%)	6 (22.2%)	

BMI: body-mass-index; BP: blood pressure; GDS: Geriatric depression scale

Cognitive impairment was assessed via the Arabic Mini-Mental State Examination-2 (MMSE-2), and scores were categorized as normal [24–30], mild [19–23], moderate [10–18], and severe (0–9) cognitive impairment

Categorical variables were summarized using frequencies and percentages, tallied by column for the overall study sample and by row for the stratified analysis by cognitive function

Continuous normal variables were summarized using mean (SD), and non-normal variables were summarized using median [IQR]

Categorical variables were assessed using a chi-square test of independence

Continuous variables with a normal distribution were compared using unpaired t-tests and one-way ANOVA

Continuous variables with a non-normal were compared using the Mann-Whitney U test and the Kruskal-Wallis test

Among lifestyle and nutrition variables, physical activity was significantly associated with MMSE status, as a higher proportion of those who were physically inactive had abnormal MMSE scores (38.8%) compared to those who were active (31.4%) (*p* = 0.048). Other lifestyle factors, including smoking, alcohol consumption, dietary intake of vitamin D and calcium, vitamin D and calcium supplementation, sun exposure, and pigmentary phototype, did not show significant differences between groups (*p* > 0.05).

Several clinical and biochemical factors were also significantly associated with abnormal MMSE scores (Table 2). Hypertension was more frequent in those with abnormal MMSE scores compared to the normal group (35.6% vs. 64.4%, *p* = 0.036). Type 2 diabetes was also associated with abnormal MMSE scores, with a higher proportion of individuals with diabetes in the abnormal group compared to the normal group (37.0% vs. 63.0%, *p* = 0.012). Geriatric Depression Scale (GDS-15) scores were higher in those with abnormal MMSE scores (8.00 [6.00;9.00]) compared to those with normal scores (7.00 [5.00;8.00]) (*p* = 0.024). Morning cortisol levels were significantly higher in the abnormal MMSE group compared to the normal group (413 ± 146 nmol/L vs. 362 ± 135 nmol/L, *p* = 0.009). Other anthropometric measures, blood pressure, comorbidities, fasting glucose, insulin, and vitamin D levels did not show significant differences between groups (*p* > 0.05).

**Table 2:** Anthropometric measures, comorbidities, laboratory and clinical data stratified by cognitive impairment

ANTHROPOMETRIC VARIABLES	Overall	Normal cognitoin		Cognitive impairment	*P*
	*N* = 253	*N* = 174		*N* = 79	
**BMI (kg/m2)**	28.8 [26.2;32.4]				
**BMI category**,** n(%)**:					0.484
Normal	31 (12.3%)	24 (77.4%)		7 (22.6%)	
Overweight	116 (45.8%)	80 (69.0%)		36 (31.0%)	
Obese	106 (41.9%)	70 (66.0%)		36 (34.0%)	
**Weight (kg)**	77.0 [68.0;86.0]	77.0 [69.0;87.0]		76.5 [67.5;81.8]	0.245
**Height (cm)**	161 [155;170]	164 [156;172]		157 [154;164]	<0.001
**Hip circumference (cm)**	109 [101;118]	109 [102;118]		110 [99.5;119]	0.583
**Waist circumference (cm)**	102 [91.3;114]	102 [92.0;114]		101 [89.0;113]	0.540
**Waist circumference risk**,** n(%)**:					0.504
Low	41 (16.3%)	26 (63.4%)		15 (36.6%)	
High	211 (83.7%)	148 (70.1%)		63 (29.9%)	
**Waist-to-hip ratio**	0.94 [0.89;0.98]	0.94 [0.89;1.00]		0.93 [0.89;0.96]	0.281
**Waist-to-hip ratio risk**,** n(%)**:					1.000
Normal	50 (19.8%)	34 (68.0%)		16 (32.0%)	
At Risk	203 (80.2%)	140 (69.0%)		63 (31.0%)	
**Vital signs and comorbidities**					
**Systolic BP (mm Hg)**	130 [123;140]	130 [123;140]		136 [129;140]	0.052
**Diastolic BP (mm Hg)**	80.0 [70.0;80.0]	80.0 [70.0;80.0]		80.0 [72.0;80.0]	0.442
**Elevated blood pressure**,** n(%)**:					0.347
No	67 (27.0%)	50 (74.6%)		17 (25.4%)	
Yes	181 (73.0%)	122 (67.4%)		59 (32.6%)	
**Comorbidities**,** n(%)**:					
a) Dyslipidemia					0.072
No	72 (28.5%)	56 (77.8%)		16 (22.2%)	
Yes	181 (71.5%)	118 (65.2%)		63 (34.8%)	
b) Hypertension					**0.036**
No	79 (31.2%)	62 (78.5%)		17 (21.5%)	
Yes	174 (68.8%)	112 (64.4%)		62 (35.6%)	
c) Thyroid disorder					0.441
No	217 (86.5%)	152 (70.0%)		65 (30.0%)	
Yes	34 (13.5%)	21 (61.8%)		13 (38.2%)	
d) Type 2 Diabetes					0.012
No	91 (36.0%)	72 (79.1%)		19 (20.9%)	
Yes	162 (64.0%)	102 (63.0%)		60 (37.0%)	
e) Cardiovascular disease					0.657
No	195 (77.4%)	132 (67.7%)		63 (32.3%)	
Yes	57 (22.6%)	41 (71.9%)		16 (28.1%)	
f) Renal disease					0.743
No	225 (88.9%)	156 (69.3%)		69 (30.7%)	
Yes	28 (11.1%)	18 (64.3%)		10 (35.7%)	
**(GDS-15) scale**	7.00 [5.00;9.00]	7.00 [5.00;8.00]		8.00 [6.00;9.00]	**0.024**
**Depression** **(GDS-15) scale categories**,** n(%)**:					**0.040**
Normal (8+)	141 (55.7%)	105 (74.5%)		36 (25.5%)	
Abnormal (< 8)	112 (44.3%)	69 (61.6%)		43 (38.4%)	
**CLINICAL & BIOCHEMICAL VARIABLES**					
**Alkaline Phosphatase (IU/L)**	69.0 [57.0;85.0]	67.0 [56.0;84.0]		77.0 [61.0;86.5]	0.043
**Phosphate (mmol/L)**	1.13 [1.03;1.22]	1.12 [1.02;1.22]		1.14 [1.06;1.22]	0.219
**Calcium (mmol/L)**	2.29 [2.24;2.37]	2.29 [2.24;2.37]		2.29 [2.23;2.37]	0.944
**Parathyroid Hormone (pmol/L)**	5.57 [4.09;7.30]	5.73 [4.47;7.12]		5.07 [3.67;7.62]	0.224
**Cortisol (morning) (nmol/L)**	377 (140)	362 (135)		413 (146)	**0.009**
**Fasting Glucose (mmol/L)**	6.13 [5.12;7.29]	6.03 [5.16;7.06]		6.46 [5.10;8.23]	0.073
**Insulin (ulU/mL)**	13.3 [8.40;18.2]	13.6 [9.00;18.4]		11.4 [7.80;17.5]	0.139
**Serum 25-OH-D level (nmol/L)**	63.0 [45.0;83.0]	63.0 [45.0;84.8]		63.0 [46.0;81.0]	0.775
**Serum 25-OH-D level (nmol/L)**:					0.708
Sufficient	90 (35.6%)	64 (71.1%)		26 (28.9%)	
Insufficient	87 (34.4%)	57 (65.5%)		30 (34.5%)	
Deficient	76 (30.0%)	53 (69.7%)		23 (30.3%)	

BMI: body-mass-index; BP: blood pressure; GDS: Geriatric depression scale

Cognitive impairment was assessed via the Arabic Mini-Mental State Examination-2 (MMSE-2)

Categorical variables were summarized using frequencies and percentages, tallied by column for the overall study sample and by row for the stratified analysis by cognitive function

Continuous normal variables were summarized using mean (SD), and non-normal variables were summarized using median [IQR]

Categorical variables were assessed using a chi-square test of independence

Continuous variables with a normal distribution were compared using unpaired t-tests and one-way ANOVA

Continuous variables with a non-normal were compared using the Mann-Whitney U test and the Kruskal-Wallis test

**Fig. 1:**
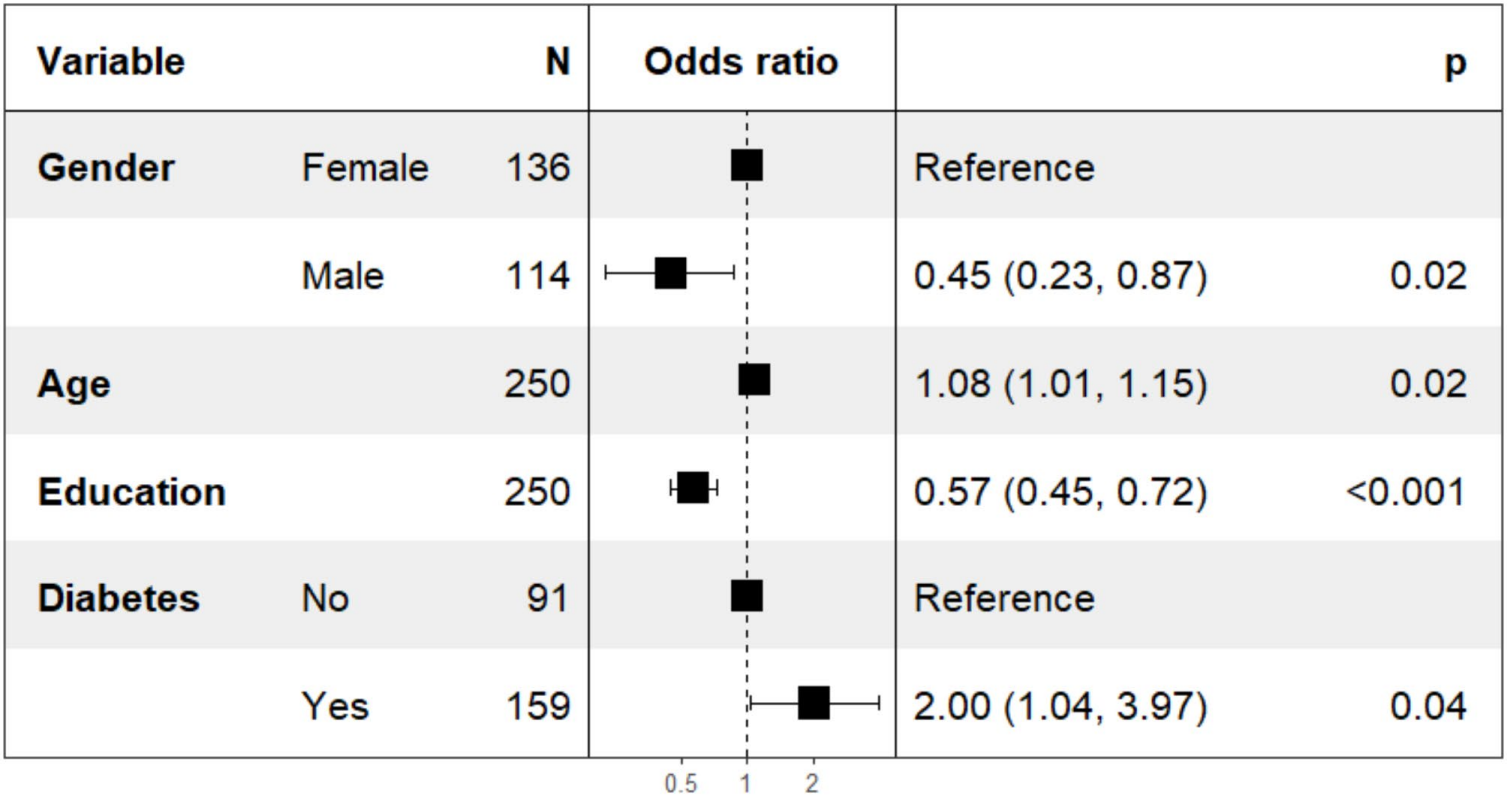
Multivariate logistic regression analysis of factors associated with cognitive impairment in older adults

The logistic regression (Fig. 1) analysis showed that several variables were associated with abnormal MMSE score variable. Males had lower odds of the having abnormal cognitive function compared to females (OR = 0.45, 95% CI: 0.23–0.87, *p* = 0.02). Age was positively associated with abnormal cognitive function, with each one-year increase in age raising the odds by 8% (OR = 1.08, 95% CI: 1.01–1.15, *p* = 0.02). Higher education was also associated with reduced odds of the abnormal cognitive function (OR = 0.57, 95% CI: 0.45–0.72, *p* < 0.001). The presence of diabetes was associated with increased odds of the abnormal cognitive function (OR = 2.00, 95% CI: 1.04–3.97, *p* = 0.04). The analysis examined the association between Mini-Mental State Examination (MMSE) classification and various anthropometric and biochemical measures, stratified by sex supplementary Table 1.

## Discussion

This study investigated the relationship between cognitive function among older adults in Kuwait and a variety of modifiable and non-modifiable risk factors spanning sociodemographic, lifestyle, nutrition, anthropometric, clinical, and biomedical variables. Our multivariate modelling, which accounted for the potential confounding effects of gender, age, body mass index category, education, physical activity, depressive symptoms, sun exposure category, alkaline phosphatase level, cortisol category, dietary calcium intake, diabetes status, dyslipidemia, showed the increased risk of cognitive function associated with Type 2 diabetes and the protective association of higher levels of education.

In this study, Type 2 Diabetes was significantly associated with higher odds of cognitive impairment, highlighting its potential role as a risk factor for cognitive decline in older adults in Kuwait. These results align with the current broader literature examining the association between Type 2 Diabetes and cognitive impairment in which there is strong consensus that diabetes is a major risk factor for cognitive impairment [43–46]. This relationship has also been found in the Middle East region. For instance, a study conducted in Saudi Arabia found that 80.3% of patients with diabetes exhibited cognitive impairment, and 33.8% exhibited severe cognitive impairment when measured with the Arabic version of the Montreal Cognitive Assessment Scale (MoCA) [45, 47]. Older age, female gender, lower education levels, and low income were associated with higher rates of cognitive decline in these patients [45]. Various potential biological mechanisms have been proposed to explain this relationship, including Type 2 Diabetes-related damage to cerebral microvasculature and macrovasculature, which are believed to contribute to cognitive decline [48, 49].Although the exact pathways are not fully clear, recent meta-analyses have suggested that regional cerebral hypoperfusion in Type 2 Diabetes is connected to a spectrum of cognitive disorders, implying that reduced cerebral blood flow in Type 2 Diabetes, possibly due to chronic inflammation, could be a potential factor in cognitive impairment [50].An additional explanation for the association between Type 2 Diabetes and cognitive function involves brain insulin resistance. It is speculated that the pathological features of diabetes, including insulin resistance, hyperglycemia, and chronic inflammation, may be associated with cognitive dysfunction [49].

This finding may be related to the univariate association between cognitive function and fasting glucose level in the present study, with higher glucose levels among those with greater cognitive impairment. Poor glycemic control has been linked to cognitive dysfunction [51].Factors such as advanced glycosylated end-product production and oxidative stress induced by hyperglycemia are suggested to contribute to neuronal and vascular endothelial damage, ultimately leading to cognitive dysfunction [51]. While the present study was cross-sectional and therefore cannot provide temporal insights, prospective research on this relationship indicates that Type 2 Diabetes typically precedes and contributes to cognitive decline [44, 46]. Maintaining optimal glycemic control is essential for preventing the complications associated with Type 2 Diabetes [44, 46, 52].

Our findings indicate that higher education levels serve as a protective factor against cognitive decline, which aligns with previous research demonstrating a similar association [53, 54]. However, while a higher educational level is associated with better cognitive function and improved quality of life in senior adults, it might not directly mitigate age-related cognitive decline [55]. Nevertheless, education can influence cognitive function in older people by helping to enhance the cognitive skills that emerge in early adulthood and persist into old age [56]. Supposing that cognitive gains reach a maximum by early adulthood, enhancing cognitive reserve and diminishing the risk of cognitive decline and dementia in later life could commence by enhancing the quality of education and broadening its accessibility during childhood and adolescence [57].

Aging is recognized as a significant risk factor for cognitive impairment, with research demonstrating an association between diminished synaptic density and cognitive decline.

The study also highlighted the significant impact of hypertension on cognitive dysfunction, with elevated systolic blood pressure being linked to more severe cognitive impairment.

The potential mechanism underlying this phenomenon is the transition of hypertension type from systolic/diastolic to systolic hypertension and aortic atherosclerosis as individuals age, significantly impacting cerebral blood supply and leading to cognitive impairment [58]. Other contributing mechanisms include oxidative stress, microvascular damage, and inflammation. Additionally, a suggested mechanism that exacerbates inflammation involves the disruption of the blood-brain barrier through microglia activation and impaired glymphatic amyloid clearance [59, 60].These latter mechanisms likely play a role in hypertension’s involvement in hastening Alzheimer’s disease processes. Therefore, efforts may be required to manage prolonged systolic blood pressure and uphold adequate diastolic blood pressure, potentially aiding in lowering the risk of cognitive impairment in older adults.

Additionally, it was found that median levels of alkaline phosphatase (ALP) and cortisol increased in direct proportion to an increase in cognitive impairment from normal to moderate/severe. This result is novel, as no prior research has explored the association between plasma ALP and cortisol levels concerning cognitive function in older adults in the Middle Eastern region.

Alkaline phosphatase has been detected on the membranes of neurons, and the activity of alkaline phosphatase in the blood (ALP) tends to rise in cases of brain injury or cerebrovascular disease. This indicates that its levels could be indicative of neuronal loss. Studies have shown that ALP levels are elevated in individuals with Alzheimer’s dementia and exhibit an inverse relationship with cognitive function [20]. From a biological perspective, associations may be explained by ALP might serve as a biomarker reflecting underlying neuroinflammatory and neurodegenerative processes. Elevated ALP levels could be linked to disrupted cellular signaling, mitochondrial dysfunction, and impaired synaptic plasticity, all of which are critical to cognitive processes [20]. However, further research is needed to fully understand the role of ALP in the cognative impairment in older adults.

Regarding cortisol levels, cortisol is likely to affect the hippocampus and prefrontal cortex. However, cortisol’s links with metabolic syndrome and neuroinflammation, as well as the disinhibition of the hypothalamic-pituitary-adrenal (HPA) axis due to neurodegeneration, are other potential mechanisms that could elucidate the link between cortisol and cognitive decline in late-life [17].Research indicates that free radicals and oxidative stress induce neuroinflammation, with neuroplasticity increasing in chronic conditions like diabetes mellitus, hypertension, and cardiovascular diseases, which elevate chronic inflammation and inflammatory markers such as C-reactive protein (CRP) and erythrocyte sedimentation rate (ESR), consequently leading to heightened cortisol levels [18, 19].

The results of this study offer insights into cognitive functioning in relation to gender differences and other related factors in older people. Cognitive impairment was found to be more prevalent in women than in men (39.1% vs. 21.7%, *P* < 0.05), with the female participants reporting higher rates of cognitive impairment and poorer global and executive function than their male counterparts. Previous studies confirm these results [61, 62]. However, other investigators have reported a higher prevalence of moderate cognitive impairment in men than in women [63, 64]. Nevertheless, the current finding shows that moderate symptoms of depression were more prevalent in the female participants. Previous studies have likewise indicated that depressive symptoms are more prevalent in women than in men [28]. By way of explanation, it has been reported that there is a connection between depression and compromised neuroplasticity in the medial prefrontal cortex and hippocampus. Additionally, memory impairments have been suggested to be linked to deficits in neuroplasticity, specifically within the hippocampus [65]. From a sociocultural perspective, cognitive impairment may be associated with gender due to cultural and educational factors in Kuwait.Late-life depression is commonly linked with cognitive decline [66].Depression and dementia exhibit overlapping and interconnected characteristics [67].Depressive symptoms are frequently observed alongside or before the onset of dementia. Furthermore, depressive disorders elevate the likelihood of persistence for mild cognitive impairment and dementia [67].Depression in older adults accelerates cognitive decline through multiple mechanisms, including hippocampal atrophy, reduced neuroplasticity, and inflammation [68].Vascular changes, cortisol dysregulation, and genetic factors also contribute to cognitive dysfunction in this population [69, 70].Chronic inflammation is a key factor in exacerbating neurodegeneration [71].In the Kuwaiti context, where the aging population is growing rapidly [4], these findings emphasize the need for early, integrated care strategies to address the growing concern of depression and cognitive decline in the elderly population, it is crucial for Kuwait to focus on early detection of depression through clinical diagnosis and screening programs. Implementing routine depression screenings in primary healthcare settings, especially for older adults, would help identify individuals at risk of both depression and cognitive decline at an early stage.

The current study revealed a negative association between physical activity and cognitive decline, highlighting physical activity as a crucial protective factor against age-related cognitive impairment. Previous population-based research has indicated that reduced levels of daily physical activity are linked to cognitive decline and an increased risk of Alzheimer’s disease in older adults [72–75]. Various potential mechanisms have been proposed in human studies to explain the beneficial impact of physical activity on cognitive function [76, 77]. For instance, physical activity may enhance cerebral blood flow by mitigating vascular risk factors [76],encouraging synaptic plasticity, and stimulating neurotrophic factors [77].Additionally, a relationship has been identified between physical activity and reduced amyloid beta peptide (Aβ) burden in positron emission tomography imaging, as well as higher levels of the 42-amino acid β amyloid peptide (Aβ42) in the cerebrospinal fluid of older adults without dementia [78].Physical activity plays a significant role in improving overall health reducing the incidence of diseases such as type 2 diabetes, hypertension, and cardiovascular conditions, which are risk factors for depression and dementia [79].Furthermore, physical activity has been associated with maintaining function and slowing decline in older adults experiencing cognitive impairment or dementia [80]. The Kuwaiti government could consider implementing a nationwide program that combines physical activity as a low-cost, non-invasive strategy that improves cognitive function, reduces dementia risk, and enhances overall health in older adults in kuwait.

These findings support the essential need for a healthy lifestyle strategy for older adults. Efforts to prevent or delay cognitive decline in Kuwait should align with strategies aimed at reducing the risk of Type 2 Diabetes. There are currently several strategies aimed at preventing diabetes and chronic disease, in general, in Kuwait, including the Kuwait National Programme for Healthy Living [81]. Continuing on this trajectory and investing in the prevention and control of non-communicable diseases in Kuwait could help mitigate the long-term burden of cognitive impairment in this aging population and is a priority of the Ministry of Health [82]. From a social determinants of health perspective, efforts can be made to encourage higher educational attainment in Kuwait. As of 2015, nearly all children attend primary school, only around 30% complete upper secondary school and less than 1% attain a Master’s degree or Equivalent in Kuwait [83]. While these figures have grown substantially over time, they indicate there is some room for improvement [83]. In addition, the importance of early screening for cognitive impairment to enable timely and appropriate management strategies cannot be overstated [84]. Earlier identification of cognitive impairment can help manage comorbidities, prevent potential safety issues, and ensure support services are available [84]. The Kuwait Ministry of Health can consider installing the mini-mental state examination (MMSE) tool in the electronic databases of primary health and geriatric clinics, connecting all public and private hospitals to facilitate diagnosis. The Ministry of Health in Kuwait can utilize these findings to implement screening programs in primary care settings, promote cognitive health awareness campaigns, and develop national policies that encourage healthy lifestyle modifications, such as increasing physical activity and dietary improvements, which have been shown to delay cognitive decline in other populations [85].

The primary strength of the present study lies in its utilization of a large, nationally representative cohort of elderly adults in Kuwait, allowing for the analysis of potential risk factors such as educational background, dietary patterns, and coexisting health conditions. This is also the first study of its kind in Kuwait, highlighting its significant contribution to understanding the health and cognitive function of the aging population in the country. The findings provide valuable insights that can guide public health initiatives and policies aimed at improving the well-being of older adults in Kuwait. However, the study is subject to several limitations. As a cross-sectional study, conclusions about causation and temporality are not appropriate. This work uses self-reported data for several variables which is subject to reporting bias. The investigation did not consider the participants’ use of medications like anti-hypertensive, anti-hyperglycemic, and lipid-lowering agents or genetic factors, which can confound the results, especially considering some drugs can affect cognition [86]. Our study did not include medications because our study did not include medications due to challenges in obtaining consistent and accurate data on medication use, which could introduce confounding variables and measurement errors. The variability in medication types, dosages, and individual treatment regimens would have complicated the interpretation of associations between biomarkers and cognitive function. We also did not have access to neuroimaging to assess stroke and brain atrophy because, including limited availability of imaging resources and the high cost associated with neuroimaging procedures. Furthermore, the large-scale nature of the study and the associated budgetary constraints made it impractical to integrate neuroimaging into the study design. Given the complex and individualized nature of neuroimaging data, incorporating it would have required additional time and resources for standardization and analysis, which were beyond the scope of this study.Additionally, the MMSE as a tool is subject to limitations including that it is a screening tool for cognitive impairment rather than a diagnostic tool, as such clinical confirmation is required to confirm a dementia diagnosis [87]. We address this limitation by explicitly referring to cognitive impairment rather than dementia in the study. The MMSE is not sensitive to mild cognitive impairment(MCI), and the cutoff scores for the MMSE vary by cultural and sociodemographic variations [88]. Consequently, while MMSE remains a useful tool for assessing overall cognitive function, it may not reliably identify subtle cognitive deficits characteristic of MCI. To address these concerns, we utilized the Arabic MMSE, which limits the risk of misclassification that would be associated with the use of the global MMSE [25]. Future efforts can focus on establishing a localized MMSE for the Kuwaiti population. The application of the average MMSE decline across the entire sample may overlook individual variations. On the positive side, the MMSE is valued for its brevity, widespread acceptance, ease of administration in clinical settings, and suitability for comparative studies across different contexts. There was an absence of data on other clinical factors that could be underlying causes of cognitive impairment (e.g., neuroimaging), and as a result, brain atrophy and stroke were not assessed as part of this study. In terms of generalizability, this study was conducted with participants already in the healthcare setting who presumably were seeking medical support for a health concern. As such, the specific results may not translate to the general older age population in Kuwait. That being said, the results coincide with other findings in this area, suggesting the plausibility that these results may be more universal and perhaps applicable to the general population and areas outside of Kuwait as well.

## Conclusions

The research findings unveiled risk factors associated with cognitive function after adjusting for potential confounders, such as Type 2 diabetes and education. While these findings align with the broader literature, conducting this study in Kuwait provides context-specific evidence that policymakers can leverage to develop targeted intervention strategies.

## Electronic supplementary material

Below is the link to the electronic supplementary material.

Supplementary Material 1

## Data Availability

The data that support the findings of this study are not openly available due to reasons of sensitivity and are available from the corresponding authors upon reasonable request.
