# Supplementary material for: Factors associated with cognitive function outcomes among older adults in Kuwait: A cross-sectional study
Source: BMC Geriatr. 2025 Apr 14;25:249. doi: 10.1186/s12877-025-05882-0 (PMC11995643; doi:10.1186/s12877-025-05882-0)
Supplement: Supplementary file 1 — Supplementary Material 1 [file 12877_2025_5882_MOESM1_ESM.docx]

**Supplementary Material**

Table 1. Association between MMSE and anthropometric measures

|  | **Normal cognition** | | | **Abnormal cognition** | | |
| --- | --- | --- | --- | --- | --- | --- |
|  | **Female** | **Male** | **P** | **Female** | **Male** | **P** |
|  | ***N=84*** | ***N=90*** |  | ***N=54*** | ***N=25*** |  |
| **BMI (Kg/m^2^)** | 30.5 [27.1;33.9] | 27.5 [25.5;30.6] | <0.001 | 30.8 [27.1;33.6] | 27.2 [26.1;28.7] | 0.025 |
| **BMI classification:** |  |  | 0.006 |  |  | 0.006 |
| Normal | 8 (9.52%) | 16 (17.8%) |  | 6 (11.1%) | 1 (4.00%) |  |
| Overweight | 32 (38.1%) | 48 (53.3%) |  | 18 (33.3%) | 18 (72.0%) |  |
| Obese | 44 (52.4%) | 26 (28.9%) |  | 30 (55.6%) | 6 (24.0%) |  |
| **Hip circumference (CM)** | 108 [101;118] | 109 [102;117] | 0.964 | 105 [98.6;117] | 114 [106;120] | 0.054 |
| **Waist circumference (CM)** | 102 (17.9) | 102 (16.4) | 0.962 | 95.7 [86.5;112] | 108 [99.0;116] | 0.058 |
| **Waist classification:** |  |  | 0.001 |  |  | 1.000 |
| Low | 4 (4.76%) | 22 (24.4%) |  | 10 (18.5%) | 5 (20.8%) |  |
| High/Very high | 80 (95.2%) | 68 (75.6%) |  | 44 (81.5%) | 19 (79.2%) |  |
| WHR | 0.94 (0.08) | 0.95 (0.08) | 0.403 | 0.93 [0.89;0.96] | 0.94 [0.89;0.96] | 0.416 |
| WHR: |  |  | 0.265 |  |  | 0.387 |
| Normal | 13 (15.5%) | 21 (23.3%) |  | 9 (16.7%) | 7 (28.0%) |  |
| At Risk | 71 (84.5%) | 69 (76.7%) |  | 45 (83.3%) | 18 (72.0%) |  |
| **Insulin ulU/mL** | 13.5 [8.97;18.0] | 13.7 [9.05;18.6] | 0.808 | 11.5 [8.05;17.0] | 11.0 [7.00;17.9] | 0.874 |
| **Ca mmol/L** | 2.31 [2.26;2.38] | 2.29 [2.22;2.35] | 0.135 | 2.31 [2.26;2.38] | 2.27 [2.22;2.32] | 0.068 |
| **GDS-15 scale** | 7.00 [5.75;9.00] | 7.00 [5.00;8.00] | 0.070 | 8.00 [7.00;9.00] | 7.00 [5.00;8.00] | 0.044 |
| **GDS-15 (Categorical)** |  |  | 0.026 |  |  | 0.131 |
| Normal (< 8) | 43 (51.2%) | 62 (68.9%) |  | 21 (38.9%) | 15 (60.0%) |  |
| Abnormal (8+) | 41 (48.8%) | 28 (31.1%) |  | 33 (61.1%) | 10 (40.0%) |  |
| **Vitamin D status:** |  |  | 0.003 |  |  | 0.644 |
| Not supplemented | 39 (46.4%) | 63 (70.0%) |  | 30 (55.6%) | 16 (64.0%) |  |
| Supplemented | 45 (53.6%) | 27 (30.0%) |  | 24 (44.4%) | 9 (36.0%) |  |
| **Vitamin D status:** |  |  | 0.004 |  |  | 0.135 |
| Deficiency | 16 (19.0%) | 37 (41.1%) |  | 12 (22.2%) | 11 (44.0%) |  |
| Insufficiency | 29 (34.5%) | 28 (31.1%) |  | 23 (42.6%) | 7 (28.0%) |  |
| Sufficiency | 39 (46.4%) | 25 (27.8%) |  | 19 (35.2%) | 7 (28.0%) |  |
| **Serum 25-OH-D level** | 53.5 [36.8;77.8] | 57.0 [42.0;78.0] | 0.712 | 73.0 [55.0;90.0] | 64.5 [53.2;81.8] | 0.334 |
| GDS: Geriatric depression scale, WHR: Waist to hip ratio  Categorical variables were summarized using counts and percentages.  Continuous normal variables were summarized using mean (SD) and non-normal variables were summarized using median [IQR]  Analysis was performed using Chi-square test of independence for categorical variables.  Continuous normal and non-normal variables were compared using one-way ANOVA and Kruskal-Wallis tests | | | | | | |

The analysis examined the association between Mini-Mental State Examination (MMSE) classification and various anthropometric and biochemical measures, stratified by sex. Among individuals with normal cognition, females had a significantly higher BMI compared to males (30.5 [27.1;33.9] vs. 27.5 [25.5;30.6], p < 0.001). A similar pattern was observed among those with abnormal cognition, where females also had a higher BMI than males (30.8 [27.1;33.6] vs. 27.2 [26.1;28.7], p = 0.025). BMI classification differed significantly across sexes for both cognition groups (p = 0.006). Among those with normal cognition, obesity was more prevalent in females (52.4%) compared to males (28.9%), whereas males had a higher prevalence of overweight status (53.3%) than females (38.1%). A similar trend was noted in individuals with abnormal cognition, where females exhibited a higher obesity rate (55.6%) than males (24.0%). For depressive symptoms, Geriatric Depression Scale (GDS-15) scores did not differ significantly between sexes in the normal cognition group (p = 0.070), but females in the abnormal cognition group had slightly higher scores than males (8.00 [7.00;9.00] vs. 7.00 [5.00;8.00], p = 0.044). GDS classification differed significantly by sex in the normal cognition group (p = 0.026), with a lower proportion of females classified as having normal GDS scores (<8) compared to males (51.2% vs. 68.9%). Among those with abnormal cognition, no significant sex differences in GDS classification were observed (p = 0.131).
